# A Novel Insight into Dehydroleucodine Mediated Attenuation of *Pseudomonas aeruginosa* Virulence Mechanism

**DOI:** 10.1155/2015/216097

**Published:** 2015-11-16

**Authors:** S. Mustafi, M. L. Veisaga, L. A. López, M. A. Barbieri

**Affiliations:** ^1^Department of Biological Sciences, Florida International University, Miami, FL 33199, USA; ^2^Biomolecular Sciences Institute, Florida International University, Miami, FL 33199, USA; ^3^Laboratory of Cytoskeleton and Cell Cycle, Institute of Histology and Embryology, Faculty of Medicine, National University of Cuyo, 5500 Mendoza, Argentina; ^4^Fairchild Tropical Botanic Garden, 10901 Old Cutler Road, Coral Gables, FL 33156, USA; ^5^International Center of Tropical Botany, Florida International University, Miami, FL 33199, USA

## Abstract

Increasing resistance of *Pseudomonas aeruginosa* (*P. aeruginosa*) to conventional treatments demands the search for novel therapeutic strategies. In this study, the antimicrobial activity of dehydroleucodine (DhL), a sesquiterpene lactone obtained from *Artemisia (A.) douglasiana*, was screened against several pathogenic virulence effectors of *P. aeruginosa*. *In vitro*, minimum inhibitory concentration of DhL was determined against *P. aeruginosa* strains PAO1, PA103, PA14, and multidrug resistant clinical strain, CDN118. Results showed that DhL was active against each strain where PAO1 and PA103 showed higher susceptibility (MIC 0.48 mg/mL) as compared to PA14 (MIC 0.96 mg/mL) and CDN118 (MIC 0.98 mg/mL). Also, when PAO1 strain was grown in the presence of DhL (MIC_50_, 0.12 mg/mL), a delay in the generation time was noticed along with significant inhibition of secretory protease and elastase activities, interruption in biofilm attachment phase in a stationary culture, and a significant decline in Type III effector ExoS. At MIC_50_, DhL treatment increased the sensitivity of *P. aeruginosa* towards potent antibiotics. Furthermore, treatment of *P. aeruginosa* with DhL prevented toxin-induced apoptosis in macrophages. These observations suggest that DhL activity was at the bacterial transcriptional level. Hence, antimicrobial activity of DhL may serve as leads in the development of new anti-*Pseudomonas* pharmaceuticals.

## 1. Introduction

*Pseudomonas aeruginosa* (*P. aeruginosa*) is a Gram-negative opportunistic pathogen with a high prominence of intrinsic antibiotic resistance [1]. Resistant strains of* P. aeruginosa* are commonly found as a secondary infection in immune-compromised patients with cystic fibrosis, COPD, AIDS, and cancer and even among diabetics [2–4], leaving serious blood stream infection with significant mortality and healthcare costs [5]. The success in establishing* P. aeruginosa* pathogenicity is largely due to formation of intractable biofilm and secretion of myriads of virulent factors including LasA protease, LasB elastase, pyocyanin, pyoverdin, Type III secretion (T3S) effectors, and alginate [6–9]. Unfortunately, selection of the most appropriate antibiotic is complicated due to the ability of* P. aeruginosa* to develop resistance to multiple classes of antibiotics.

*P. aeruginosa* can develop resistance to antibiotics because of the low permeability of its outer membrane, the constitutive expression of various efflux pumps [10], and the naturally occurring chromosomal AmpC *β*-lactamase, turning it resistant toward penicillin G, aminopenicillins, and first- and second-generation cephalosporin [11].* P. aeruginosa* easily acquires additional resistance mechanisms, which leads to serious therapeutic problems [12]. Currently, antipseudomonas treatments include higher-than-usual doses of *β*-lactam, fluoroquinolones, and amino glycosides, which possess a high degree of toxicity and very low eradication rate [13]. The dearth of successful antibiotics to completely control* P. aeruginosa* infection makes it crucial to finding alternatives to currently available drugs. Since the pathogenicity of* P. aeruginosa* is regulated by several secretory-systems mediated cell-to-cell communications, inhibition of this system can cause attenuation of virulence and protect against infection [14, 15].

Artemisia, the largest diverse genera of Asteraceae family, possesses medicinally valuable essential oils and secondary metabolites [16, 17]. Many studies indicate antimicrobial activity in* Artemisia (A.) *spp. [18–20].* A. douglasiana* is well documented as preventive folk medicine in Argentina [21]. Dehydroleucodine (DhL), a sesquiterpene lactone of the guaianolide group, is one of the principal active secondary metabolites in* A. douglasiana* [21]. DhL, first isolated from* Lidbeckia pectinata* [22], possesses cytoprotective activity [23] as well as antimicrobial activity against several pathogens [24–27].

In this study, we tested the antibacterial activity of DhL and against* P. aeruginosa* virulence. At first we evaluated the minimum inhibitory concentration (MIC) and its synergistic effect on other commercial antibiotics, and then we examined the antipseudomonal properties of this phytochemical in terms of arresting growth and attenuating virulent factors such as biofilm formation and secretion of Type III effectors, elastase A, and elastase B. We also assessed the capacity of this phytocompound to inhibit* P. aeruginosa* induced host cell toxicity.

## 2. Material and Methods

### 2.1. Reagents

Dehydroleucodine was extracted from* A. douglasiana* (Voucher specimen 2012-1018-F, Fairchild Tropical Botanical Garden, Miami, FL) as previously documented [21, 28]. DH-DhL was prepared as described previously [21]. All chemicals and reagents were purchased from Sigma-Aldrich (St. Louis, MO), unless otherwise indicated. Primary and secondary antibodies used in immunoblotting were purchased from Cell Signaling Technology Inc. (Danvers, MA). Culture supplies were purchased from Invitrogen Life Technologies (Carlsbad, CA). Prototypic* P. aeruginosa *strains used in this study are PAO1, clinical isolates PA103 and PA14 (kindly provided by Dr. Dara Frank, University of Wisconsin, Madison), and* P. aeruginosa* strain PA103ΔUΔT, expressing pUCP plasmid-encoded ExoS tagged with a hemagglutinin epitope (ExoS-HA) (kindly provided by Dr. Joan C. Olson, West Virginia University). In addition,* Staphylococcus aureus* (ATCC 12600) was used in the LasA assay. Cells were maintained in Luria Broth (LB) (Life Technologies, Carlsbad, CA) or* Agrobacterium* (AB) minimum medium (Bio-World, Visalia, CA) to which glucose and casein amino acids [20%, wt/vol] were added.

### 2.2. Determination of Minimum Inhibitory Concentration (MIC) and Minimum Bactericidal Concentration (MBC)

The minimum inhibitory concentration of DhL was determined by micro dilution broth assay [12] for PA01, PA103, PA14, and CDN118 strains of* P. aeruginosa*. Serial doubling dilution of DhL was made, ranging from 2.24 mg/mL to 0.224 *μ*g/mL. Twenty (20) *μ*L of standard inoculums (0.5 McFarland) of all three* P. aeruginosa* strains was introduced to desired volume of growth medium and incubated at 37°C for 18 hr. Controls were set up as follows: (1) sterility control: LB broth only; (2) growth control (negative): LB broth + bacteria; (3) positive control: LB broth with Gentamycin; (4) vehicle control: LB broth + bacteria + DMSO. MIC was interpreted as the least concentration with no observable turbidity. Optical density readings (*λ* = 600 nm) were taken using microplate reader at 0 and 18 hr. MIC_90_ and MIC_50_ were determined as the concentration of DhL that inhibited bacterial growth by 90% and 50%, respectively. Results were reported as the MIC, MIC_50_, and MIC_90_ for growth at 18 hr after inoculation.

Minimum bactericidal concentration (MBC) was recorded as a lowest extract concentration killing 99.9% of the bacterial inocula after 24 h incubation at 37°C. Each experiment was repeated at least three times. MBC values were determined by removing 100 *μ*L of bacterial suspension from subculture demonstrating no visible growth and inoculating nutrient agar plates. Plates were incubated at 37°C for a total period of 24 h. The MBC is determined with the wells whose the concentrations are ≥ MIC [29]. According to the ratio MBC/MIC, we appreciated antibacterial activity. If the ratio MBC/MIC is 1 or 2, the effect was considered bactericidal but if the ratio MBC/MIC is 4 or 16, the effect was defined as bacteriostatic [30].

For each assay 0.12 mg/mL (MIC_50_) of DhL was added to* P. aeruginosa* culture at early log phase unless otherwise stated. The effect of DhL on bacterial cell proliferation was determined by monitoring the growth curve of* P. aeruginosa* strain PAO1. Briefly, an overnight culture (in LB medium) of PAO1 was diluted to OD_600_ 0.05 in LB medium (control) or LB and DhL (0.12 mg/mL) and incubated at 37°C while shaking. The OD_600_ was monitored at 30 min intervals until OD_600_ of approximately 1.7 was obtained (approximately 8 hr). All OD_600_ measurements were verified at a 1/10 dilution for greater accuracy.

### 2.3. Determination of Antibiotic Synergy with DhL by the E-Test Strip

Bacterial suspensions (PAO1) homogenized in sterile saline were prepared from overnight fresh cultures to a McFarland standard of 0.5 and were spread with a sterile cotton swab on 150 mm Mueller-Hinton agar control and containing DhL (MIC_50_) plates. The agar plates were allowed to stand for 15–20 min at room temperature to allow any excess surface moisture to be absorbed before placement of MIC test strips. Antibiotic gradient strips (E-test, Liofilchem, Italy) containing Gentamycin (concentration range 0.016–256 mg/L), Ciprofloxacin (concentration range 0.002–32 mg/L), or Chloramphenicol (concentration range 0.016–256 mg/L) were then used; the MHA plates with the over layered strips were incubated at 37°C or 24 hr, and the growth inhibition zones were measured. Differences in MIC values between the control and test plates were recorded (end points were determined according to the manufacturer's instructions).

The Muller Hinton agar (MHA) dilution method was used to evaluate the Fractional Inhibitory Concentration Index (FICI) of DhL with Chloramphenicol on PAO1 being tested following previously established protocol [31, 32]. Eight serial twofold dilutions of DhL were prepared as described before, to obtain final concentration range of 2.24 mg/mL–0.224 *μ*g/mL. A series of twofold serial dilutions of Chloramphenicol was also prepared similar to DhL. All antibacterial standards dilutions were mixed with the appropriate concentration of DhL compounds thus obtaining a series of the combinations of Chloramphenicol and DhL. The concentrations prepared corresponded to 1, 1/2, 1/4, 1/8, 1/16, 1/32, 1/64, 1/128, and 1/256 of MIC values. The 96-well plate containing 100 *μ*L of Mueller Hinton (MH) broth was used. For bacteria strain PAO1, three columns of eight wells of 96-well plate were used. Each well received the culture medium + combination of DhL with Chloramphenicol + inoculum (10 *μ*L of inocula) and INT (sigma-Aldrich, St. Louis, MO) (50 *μ*L; 0.2 mg/mL). The plates were covered and incubated at 37°C for 24 hr. All tests were performed in triplicate and the bacterial activity was expressed as the mean of inhibitions produced. Inhibition of bacterial growth was judged by rose or yellow color. The analysis of the combination of DhL and Chloramphenicol was obtained by calculating the Fractional Inhibitory Concentration Index (FICI) as follows: FICI = (MIC of the combination of DhL and Chloramphenicol/MICa alone) + (MIC of the combination of DhL and Chloramphenicol/MICb alone), where MICa is Minimal Inhibitory Concentration of DhL and MICb is Minimal Inhibitory Concentration Chloramphenicol. The FICI was interpreted as follows: (1) a synergistic effect when FICI ≤ 0.5; (2) an additive or indifferent effect when FICI > 0.5 and <1; and (3) an antagonistic effect when FICI > 1.

### 2.4. LasA and LasB Activities

Staphylolytic activity of LasA was determined spectrophotometrically by measuring the decrease in absorbance at 595 nm (OD_595_) due to lysis of heat inactivated* Staphylococcus aureus* (*S. aureus*) cells (0.3 mg/mL; 0.02 M Tris-HCl, pH 8.5) upon addition of PAO1 culture supernatants [34]. One hundred (100) *μ*L aliquot of* P. aeruginosa* culture supernatant with or without DhL (after normalizing the supernatant total protein concentration) was added to 900 *μ*L of a boiled* S. aureus* suspension. The OD_595_ was determined after 0, 5, 10, 20, 30, 45, and 60 min, respectively. Activity was expressed as the change in the OD_595_/hr/*μ*g protein. One unit of activity was defined as the amount of enzyme that causes OD_595_ decrease of 1 absorbance unit/min.

Elastolytic activity of LasB in PAO1 culture fluids was determined by elastin Congo red (ECR) assay [35]. One hundred (100) *μ*L aliquot of the AB medium culture supernatants from mid log phase with or without DhL treatment (concentration was normalized) was added to 900 *μ*L of ECR buffer (100 mM Tris-HCl, 1 mM CaCl_2_, pH 7.5) containing 20 mg ECR. Tubes were incubated for 18 hr at 37°C with rotation and then were placed on ice after 0.1 mL of 0.12 M EDTA was added. Insoluble ECR was removed by centrifugation, and the OD_495_ was measured. Absorption due to pigments produced by* P. aeruginosa* was corrected for by subtracting the OD_495_ of each sample that had been incubated in the absence of ECR. Cell-free AB medium alone and AB medium with DhL were used as negative controls.

### 2.5. Relative Gene Expression Analysis of LasR

Total mRNA was purified from mid log phase PAO1 (10^8^ cells/mL) untreated or after DhL treatment using QIAGEN RNeasy mini kit (QIAGEN, Valencia, CA) and quantified following standard techniques. cDNA was synthesized from 1 *μ*g of mRNA using iScript Supermix (Biorad, Hercules, CA). Real time PCR was performed in triplicate from each sample using a Biorad CFx96 Real Time System (Biorad, Hercules, CA) and SsoAdvanced SYBR Green Supermix (Biorad, Hercules, CA) with the primers for LasR (5′-GA AAT GTG CCT TTC CGG CAC AAC-3′ and 5′-AGG CCA TAG CGC TAC GTT CTT CTT-3′) and gyrB (DNA-gyrase subunit B: 5′-TGC TGA AGG GGC TGG ATG CCG TAC GCA AGC-3′ and 5′-TAT CCA CCG GAA TAC CGC GTC CAT TGT CGC-3′). Relative gene expression was determined using the 2^−ΔΔC_T_^ method [36]. Mean C_T_ of triplicate measures was computed for each sample. Sample mean C_T_ of gyrB (internal control) was subtracted from the sample mean C_T_ of the respective gene of interest (ΔC_T_). The ΔC_T_ of the sample with no treatment was selected calibrator and subtracted from the mean ΔC_T_ of each experimental sample (ΔΔC_T_). 2^−ΔΔC_T_^ yields fold change in gene expression of the gene of interest normalized to the internal control gene expression.

### 2.6. Polyvinyl Chloride Biofilm Formation

The effect of DhL on the attachment phase of biofilm formation was measured by using the polyvinyl chloride biofilm formation assay [37]. Briefly, overnight cultures of PAO1 were resuspended in fresh AB medium in the presence and the absence DhL. After 24 hr of incubation at 30°C, the biofilms in the polyvinyl chloride microtiter plates were visualized by staining with a crystal violet solution. Plates were rinsed once to remove planktonic cells, and then attached cells to the surface were quantified by solubilizing the dye in ethanol by measuring the absorbance at OD_546_.

### 2.7. Type III Secretion Assays

Low calcium fractionation protocol was adopted for the detection of Type III secretion effectors [8]. Briefly,* P. aeruginosa* PAO1, PA103ΔUΔT (control), and PA103ΔUΔT expressing HA-ExoS were grown overnight in LB. Bacteria were then subcultured 1 : 1,000 in LB supplemented with 5 mM EGTA with or without DhL and grown for 6 h at 37°C with aeration. Bacterial densities were determined at OD_600_. Bacteria were sedimented by centrifugation at 3,220 ×g for 15 min at 4°C.

Culture supernatant was collected, and proteins were precipitated with 5% trichloroacetic acid and washed once with ice-cold acetone. Proteins were resuspended according to culture density and separated by SDS-PAGE. Proteins derived from precipitated PAO1 supernatant were run on SDS-PAGE and then either stained with Coomassie blue or transferred onto nitrocellulose membranes as described below.

### 2.8. Immunoblotting Analysis

Samples (treated and nontreated with DhL) were resuspended in sample buffer and then separated by SDS-PAGE, transferred onto nitrocellulose membranes (Biorad Laboratories, Hercules, CA, USA), and incubated with antibodies specific to HA-epitope tag for 2 hr at room temperature. Subsequently, membranes were incubated with the appropriate horseradish peroxidase-conjugated secondary antibodies. A Super Signal West Pico chemiluminescent substrate kit (Pierce/Thermo Fisher Scientific, Inc., Rockford) was used to visualize protein bands. Band densities from Western blots were quantified using the Image J64 software at a gray-scale amplification of 600 dpi.

### 2.9. *P. aeruginosa* Mediated Apoptosis of Macrophage Cells

The effect of DhL treatment on* P. aeruginosa* mediated apoptosis of J774-Eclone mouse macrophage cell was monitored as previously described [38]. J774-Eclone cell monolayer was plated from suspension culture 1 day prior to infection in DMEM supplemented with 5% FBS (DMEM-5%FBS). Cell monolayer (5 × 10^5^ cells per well; >80% confluence) was washed with phosphate-buffered saline (PBS), mixed with either DhL treated or DhL-untreated PAO1 bacteria at a multiplicity of infection (MOI) of 20 and incubated for 2 hr at 37°C in a 5% CO_2_ incubator. The cells were washed with PBS to remove the nonadhering bacteria. Fresh DMEM containing 5% FBS was supplemented with 400 *μ*g/mL of Gentamycin and then added to cells for an additional 2 hr to kill any unwashed* P. aeruginosa*. As positive control for the apoptosis, J774-Eclone cells were incubated with 0.1 *μ*M of Staurosporine (Fischer Scientific, Waltham, MA) for 1 hr. Cells were washed once with PBS and stained with 1 mg/mL Hoechst 33258 (Molecular Probes, Inc., Eugene, OR) for 10 min in the dark as described in Section 2. Chromatin condensation was examined under the fluorescence microscope by using a DAPI (4, 6-diamidino-2-phenylindole) filter after stained cells were mounted onto slides using mounting medium.

### 2.10. Statistical Analysis

All experiments were performed independently in triplicate and each experiment was repeated three times. Values represent the mean ± SEM of three independent experiments. Data were analyzed by Mann-Whitney* U* test with a significance threshold value of 0.05, by using the SPSS (Chicago, IL) statistical software package.

## 3. Results

We first examined the antibacterial effects of DhL against several* P. aeruginosa* strains (PAO1, PA103, PA14, and clinical strain CDN118, a multidrug resistant strain) with varying concentrations of DhL ranging from 2.24 mg/mL to 0.224 mg/mL. As displayed in Table 1, DhL inhibited 50% of PAO1 and PA103 population at 0.12 mg/mL whereas 0.48 mg/mL of DhL successfully inhibited 50% of PA14 population. The minimum inhibitory concentration that is the lowest concentration of the DhL solution required to completely inhibit the growth of the* P. aeruginosa* was 0.48 mg/mL for PAO1 and PA103 as compared to 0.96 mg/mL for the more virulent strain PA14 (Table 1). However, for the clinical strain DhL MIC was 0.28 mg/mL (Table 1). The bactericidal and bacteriostatic effect of DhL was determined by MBC/MIC (Table 2). The antimicrobial effect of DhL is prominent against all strains of* P. aeruginosa* tested here, although strains PAO1 and PA103 seemed to be more susceptible to DhL than PA14 and clinical strain CDN118.

**Figure 1:**
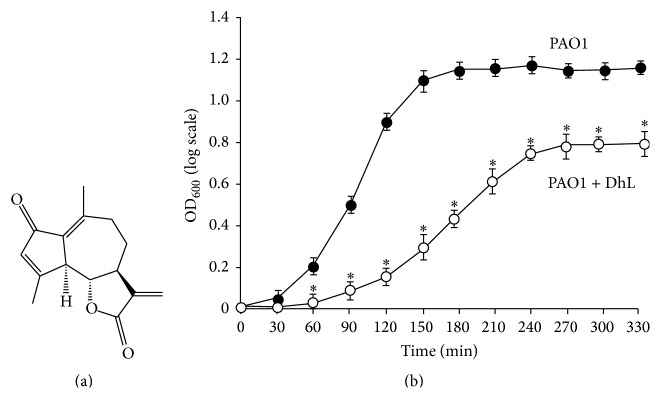
DhL inhibits growth of* P. aeruginosa*. (a) Chemical structure of DhL. (b)* P. aeruginosa* PAO1 strain was grown in the absence (○) and presence (●) of 0.12 mg/mL DhL from early log phase to stationary phase (1.7 hr) at 37°C with constant shaking. Generation time (between 1 and 3 hr) of PAO1 in presence of DhL (calculated from three independent experiments) was 64 ± 5 min whereas it was 35 ± 5 min in the control PAO1 culture. Data represent the mean of three independent experiments ± SEM.

We then determined whether DhL activity at MIC_50_ concentration was effective to slow down the bacterial growth* in vitro* in a time-dependent manner. We found that the ideal growth phases of PAO1 represented a log phase during the first 150 min and then a stationary phase with OD_600_ 1.7 that remained until 360 min (Figure 1). Treatment with DhL at MIC_50_ concentration (0.12 mg/mL) declined the slope of log phase that ended at 270 min of culture, which then turned to stationary phase at a very low OD_600_ (i.e., 0.7). Noticeable difference in generation time (50 ± 5%) was observed between 1 and 3 hr of growth (mid log phase) between treated and untreated PAO1 (Figure 1). Treatment at MIC_90_ failed to portray normal growth curve (data not shown).

**Figure 2:**
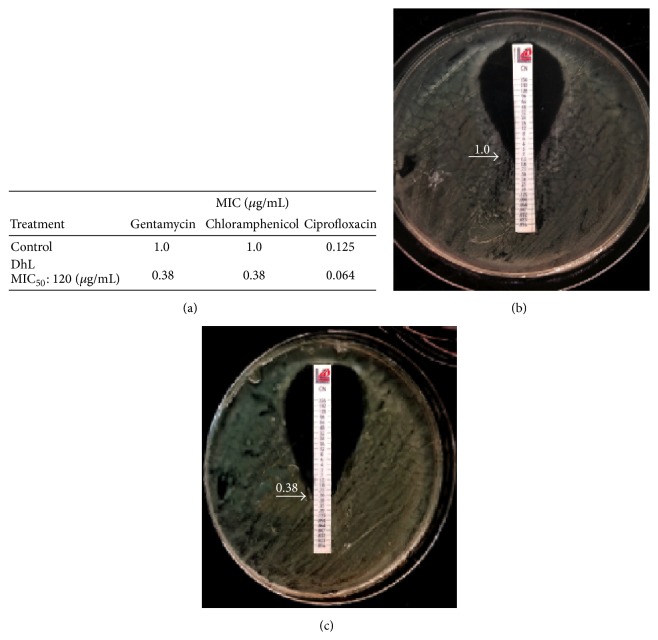
Synergistic effect of DhL with systemic antibiotics. (a) Table represents synergistic activity of subinhibitory concentration of DhL and antibiotics belonging to various classes determined by the E-test strip/agar dilution method against PAO1. (b) A control plate (MHA) with Chloramphenicol E strip. (c) A Chloramphenicol E-test plate (MHA) with DhL at a dilution of MIC_50_ concentration.

Several studies have suggested that combining plant- or animal-derived natural compounds with antibiotics is a new strategy for developing therapies against infections [39–41]. We report that the antimicrobial activities of commercial antibiotics are enhanced by pretreating* P. aeruginosa* with 0.12 mg/mL DhL. MIC values were decreased by the coactions of DhL with gentamicin (aminoglycoside), Chloramphenicol and Ciprofloxacin (fluoroquinolone), Figure 2. To further confirm the combined effect of DhL and conventional antibiotics we calculated the FICI for DhL and Chloramphenicol combination on PAO1 (Table 3). Overall the observations from both assays demonstrated significant antibacterial synergy activities of DhL on* P. aeruginosa*.

**Figure 3:**
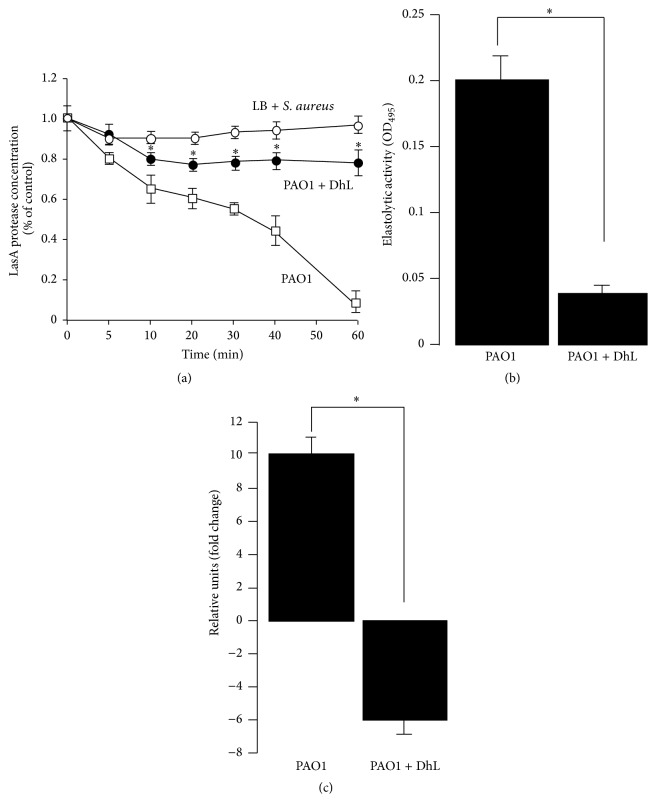
Effect of DhL on* P. aeruginosa* strain PAO1 protease activities. Staphylolytic LasA and elastolytic LasB activities were monitored in the absence or in the presence of DhL (0.12 mg/mL). (a) To measure the proteolytic activity of LasA within the supernatant fraction of the PAO1 growth culture, a staphylolytic assay was employed accounting the lysis of heat inactivated intact* S. aureus* at OD_595_ as described in Section 2. Proteolytic activity of PAO1 supernatant (PAO1, □) was significantly (asterisk indicates *P* < 0.05) inhibited when PAO1 were treated with DhL (PAO1 + DhL, ●). LB +* S. aureus* was used as control (○). Data represent the mean of three independent experiments ± SEM. (b) Levels of LasB elastolytic activity (within the PAO1 cultural supernatant with or without DhL treatment) were measured by Elastin Congo red assay at OD_495_ as described in Section 2. PAO1 were grown in LB broth at 37°C for 16 hr. The cultures were adjusted to an OD_540_ of 3.5–4.0 before harvesting to eliminate growth-related variations in elastolytic activity. Significant (asterisk indicated *P* < 0.05) decrease in LasB activity was observed in PAO1 when treated with DhL. Data represent the mean of three independent experiments ± SEM. (c) Relative mRNA expression level of LasR gene at mid log phase from DhL treated* P. aeruginosa* shows fivefold inhibition compared to untreated population. Data represent the mean of three independent experiments ± SEM.

Elastase A and elastase B (LasB) are effectors of Type II secretion system in* P. aeruginosa* and are major virulent factors in lung infection, often related to high death rate in acute pneumonia [42]. The elastase properties of LasA and protease activity of LasB play a major role in* P. aeruginosa* pathogenesis [43, 44]. LasB secretion is a prominent virulence factor in cystic fibrosis lung infection [45], which is known to degrade elastin and collagen in host [46]. To investigate the effect of DhL on LasA and LasB activities,* P. aeruginosa* strain PAO1 was incubated with DhL at 0.12 mg/mL (MIC_50_) as described in Section 2. PAO1 cultural supernatant showed an exponentially increasing staphylolytic effect indicating protease activity when compared to* S. aureus* concentration in LB as a control (Figure 3(a)). A significant decrease (89 ± 3%) in LasA activity (Figure 3(a)) was observed after 60 min of incubation of* S. aureus* substrate along with the DhL treated* P. aeruginosa* strain PAO1. Furthermore, significant decrease of 75 ± 5% in LasB activity was also observed when PAO1 was grown in the presence of DhL (Figure 3(b)). In parallel control studies, DhL along with* S. aureus* in absence of PAO1 did not show any LasA activity as well as DhL and LB alone did not show elastase activity (data not shown). In both experiments, PAO1 cultures grown in presence or absence of DhL were normalized so that the total number of bacteria remained the same.

LasR is a transcription regulator of LasA and LasB gene [43, 47]. The mRNA expression level of LasR in PAO1 population from mid log phase reduced fivefold after DhL treatment (Figure 3(c)). These results indicated DhL effectively inhibited both LasA and LasB activity in PAO1 population by controlling transcription regulator in live bacteria, and the absence of such virulent activities was not due to loss of bacterial viability.

**Figure 4:**
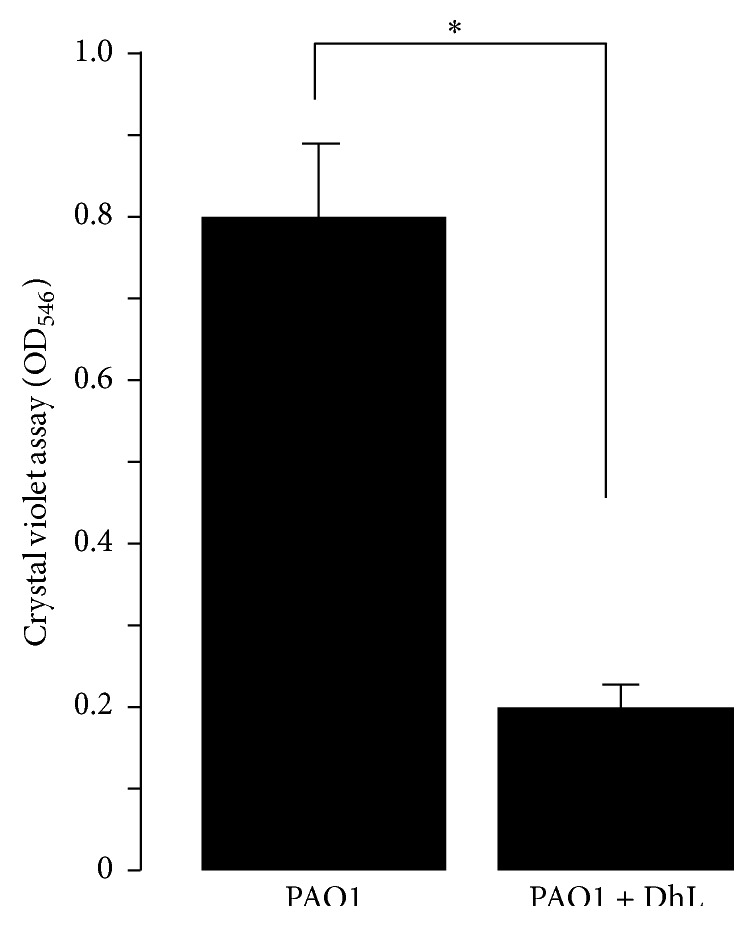
Effect of DhL on* P. aeruginosa* biofilm formation. Overnight cultures of PAO1 were resuspended in fresh AB medium (control) and in AB medium supplemented with DhL (see Section 2) in PVC tubes. After 24 hr of incubation at 30°C, the biofilms on the wall of the tubes were visualized by staining with a crystal violet solution. Biofilm formation was quantified by measuring OD_546_ of crystal violet-stained wells rinsed with ethanol. Each column is the mean of three individual experiments with two replicates per treatment. Significant (asterisk indicated *P* < 0.05) decrease in biofilm formation was observed in PAO1 when treated with DhL. Data represent the mean of three independent experiments ± SEM.

*P. aeruginosa* has the ability to form biofilms, in which cells are organizedinto layers and enmeshed in a matrix of mucoid polysaccharides [48].* P. aeruginosa* biofilms are linked to quorum sensing behavior [48, 49] by using two distinct acyl-homoserine lactone [3]. Biofilm-grown bacterial cells show increased resistance to antibiotics [48]. To determine whether DhL could prevent biofilm formation,* P. aeruginosa* strain PAO1 was incubated in the absence or in the presence of DhL and then the generation of biofilms was quantified as described in Section 2. Results indicated a significant decrease of 55 ± 5% in biofilm formation (Figure 4) in DhL treated PAO1 compared to the control, suggesting an inhibitory effect of DhL on* P. aeruginosa* biofilm formation.

**Figure 5:**
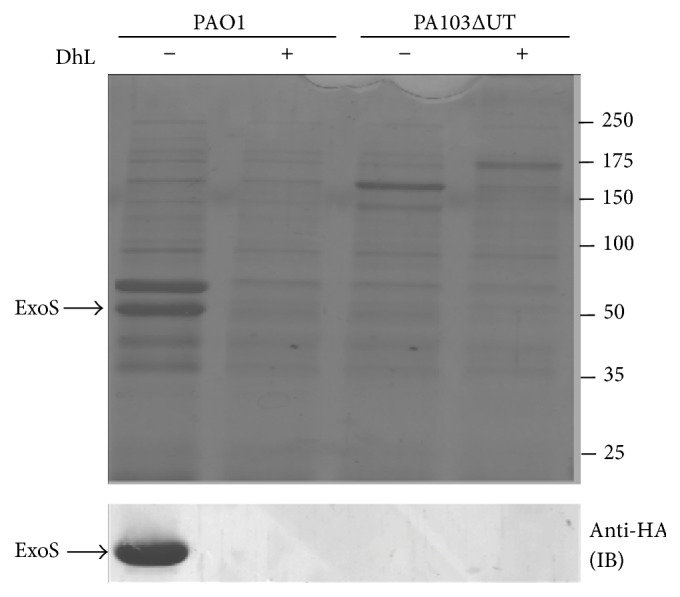
Effect of DhL on Type III secretion. Top panel represents Type III secretion profile of* P. aeruginosa* strain PAO1 in presence of DhL as observed with SDS-PAGE stained with Coomassie blue stain. Type III null mutant (PA103 ΔUT) was used as a control. Lane 1 represents untreated PAO1 secretion profile and lane 2 represents treated PAO1 secretion profile. Under Coomassie blue stain, protein bands were observed at ~53 kDa, ~49 kDa, and ~37 kDa, indicating ExoT, ExoS, and ExoY, respectively, in PAO1 Type III secretion assay, which were not shown in DhL treated PAO1 Type III secretion assay. Lanes 3 and 4 showing untreated and DhL treated control strain (PA103 ΔUΔT) with no visible Type III secretion profile. Bottom panel represents immunoblot results of DhL treated PA103ΔUΔT: HA-ExoS strain carrying HA-tagged ExoS as described in Section 2. HA specific antibody was used in immunoblot (IB) to confirm the presence of ~49 KDa ExoS secretion. Experiments were repeated three times to confirm the observation.

T3S system is a complex multiprotein apparatus that facilitates the secretion and translocation of effector proteins such as ExoS, ExoT, ExoU, and ExoY from the cytoplasm of bacterial cell to the cytoplasm of mammalian cells. Active T3S system is significantly associated with acute infections, extreme clinical outcomes, and death in patients infected with* P. aeruginosa* [50]. ExoS and ExoT are bifunctional proteins with Rho-GAP and ADP-ribosylation activity whereas ExoU is a potent phosphor lipase and ExoY is adenylate cyclase [9]. In animal infection models mimicking* P. aeruginosa* mediated acute human infections, such as burn wounds, acute pneumonia, and corneal infection, it was shown that T3S is an important virulence mechanism [51, 52]. To determine whether DhL inhibits the T3S system-mediated secretion of effector proteins such as ExoS,* P. aeruginosa* strain PAO1 was incubated in the absence or in the presence of DhL under T3S system-inducing conditions (see Section 2). The secreted effectors were analyzed on SDS-PAGE as described in Section 2. DhL inhibited the secretion of ExoS in PAO1 by at least 95% (Figure 5 (top panel)). To further confirm this result, presence of ExoS was screened in the secretion profile of PA103ΔUΔT: HA-ExoS strain, carrying HA-tagged ExoS, in the absence or in the presence of DhL. The secretion of HA-tagged ExoS was probed with immunoblotting using specific anti-HA antibody. These results indicated that DhL blocked the secretion of HA-ExoS (Figure 5 (bottom panel)).

**Figure 6:**
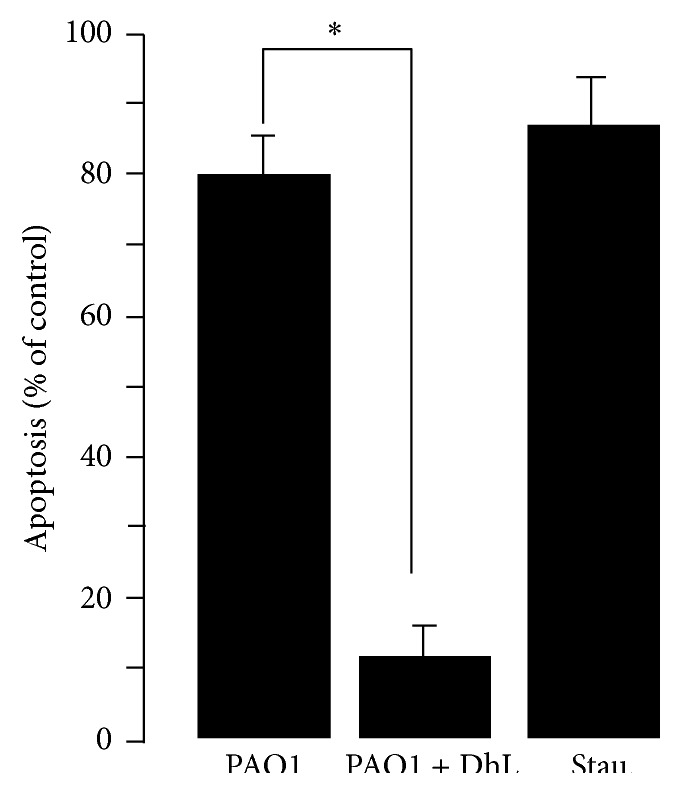
Effect of DhL on* P. aeruginosa* induced apoptosis. J774-Eclone cells were plated in monolayer (5 × 10^5^ cells per well); once adhered they were infected with PAO1 bacteria previously grown in LB-DhL (MIC_50_, 0.12 mg/mL) or only LB, at a multiplicity of infection of 20, and incubated for 2 hr at 37°C in a 5% CO_2_ incubator. In a parallel experiment an equal number of J774-Eclone cells was subjected to 1 *μ*M Staurosporine for positive control. Cells were then washed with PBS, stained with Hoechst dye, and subjected to fluorescence microscopy (Section 2). Five fields were randomly sampled from each experimental population, and all of the cells stained with Hoechst dye in each field were counted up to 500 in total. The total number of apoptotic cells with condensed or fragmented nuclei was determined in the five sampled regions and was expressed as follows: percentage of apoptosis per sample: number of apoptotic cells/total number of cells × 100. Each column is the mean of three individual experiments with two replicates per treatment. Significant (asterisk indicates *P* < 0.05) decrease in apoptotic cells was noticed when infected with PAO1 grown in presence of DhL. Data represent the mean of three independent experiments ± SEM.

Several Studies have suggested that T3S system effectors from* P. aeruginosa* are responsible for inducing rapid apoptosis in macrophage cells and epithelial cells [49]. In an effort to support our previous observation, antimicrobial activity of DhL was tested in a cellular activity assay for T3S system effector translocation into mammalian cells [53].* P. aeruginosa* PAO1 strain was added to J774-Eclone cell monolayer at MOI of 20 : 1 in the absence or in the presence of DhL as described in Section 2. After 2 hr of incubation at 37°C, cells were stained with Hoechst stain and apoptotic nuclei were observed under fluorescent microscope. The total percentage of apoptotic cell counted suggests that DhL treated* P. aeruginosa* PAO1 strain fails to induce apoptosis significantly when compared to untreated PAO1 induced apoptosis (control). Almost 87 ± 5% inhibition of apoptosis was observed in PAO1 population previously treated with DhL (Figure 6). As a positive control of apoptosis we have treated J774-Eclone cells with Staurosporine (see Section 2). Together, these results demonstrate that DhL suppresses the T3S system-mediated secretion of effectors from PAO1 as well as the translocation of effectors into the target mammalian cells.

## 4. Discussion

*P. aeruginosa* represents a wide number of strains with strong pathogenic capacity and has developed a number of strategies to affect and invade the host cell. PAO1 strain of* P. aeruginosa* is the standard laboratory strain as well as genetic reference strain [33]. PA14, a “multihost” pathogen, is capable of infecting animals (in a burned mouse model), plants, insects, and nematodes [54]. Both strains were able to initiate and maintain chronic infection in rat lung model [55]. Exotoxin-U producing strain, PA103, is a cytotoxic strain of* P. aeruginosa*, which is known to cause severe alveolar epithelial injury during infection [56]. In this study, we have demonstrated that DhL is bacteriostatic for virulent strains of* Pseudomonas aeruginosa*, PA14, and CDN118 [57, 58] but bacteriocidal for less virulent strains PAO1 and PA103 of* P. aeruginosa*.

When PAO1 strain was treated with sub-MIC dose of DhL (MIC_50_ = 0.12 mg/mL) and inoculated in growth medium, a significantly reduced (50 ± 5%) generation time was observed. Such treatment also modified the susceptibility of* P. aeruginosa* toward potent antibiotics and lowered the MIC systemic antibiotics. A combination of sub-MIC levels of DhL and antibiotics can help reduce undesirable side effects from routine antibiotic dosage. The FICI assay of DhL with conventional antibiotic on PAO1 strain showed significant synergistic effect.

DhL treatment also inhibited severalvirulence factors in PAO1 at the threshold dose of 0.12 mg/mL. PAO1 cultural supernatant treated with DhL showed 89 ± 3% decrease in LasA activity and 75 ± 5% reduction in LasB activity. Significantly low mRNA expression of transcription regulator LasR corroborates with the activity status as well. It is important to note that the virulence mechanisms in* P. aeruginosa *are highly controlled by transcription factors and regulators. It is well documented that LasA and LasB transcription activation requires* LasR* gene products [33].

DhL treated PAO1 culture failed to demonstrate successful initial attachment phase during the course of biofilm formation and prolonged observations revealed disruption in biofilm formation compared to untreated PAO1 culture (data not shown). Inhibitory effects of DhL were also poignant for* P. aeruginosa* T3S system that successfully restrained secretion of important T3S effectors such as ExoS, ExoT, ExoY, and ExoU. Overall, the results indicated a prominent regulation of* P. aeruginosa* growth and virulence due to DhL treatment* in vitro*. Finally, when DhL treated* P. aeruginosa* was cocultured with mouse macrophage cells, the typical PAO1 induced cellular apoptosis was successfully controlled, reinforcing the active inhibition of T3S system virulence which otherwise leads to cytotoxicity.

Although the exact mechanism of DhL mediated suppression of* P. aeruginosa* virulenceis still under investigation, it is likely that DhL imparts a direct effect within the bacterial cells rather than extracellular substances and mucoid layers of biofilm. Previous studies suggested that the *α*-methylene-*γ*-lactone moiety in sesquiterpene lactones like DhL exerts its biological activities by Michael-type additions attacking nucleophiles. The modified nucleophiles react reversibly with sulfhydryl groups in the cell such as transcription factors [59, 60]. To confirm the functional properties of DhL, we utilized a DhL derivative, which is a mixture of two epimers (11S)DH-DhL and (11R)DH-DhL [21]. DH-DhL epimers which are devoid of the highly reactive *α*-methylene-*γ*-lactone moiety failed to show any significant antipseudomonalactivity at similar concentration to DhL (0.24 mg/mL).

Due to high mutation rate in bacterial genome,* P. aeruginosa* is extremely difficult to eradicate from mucoid colonies [61]. Therefore, alternatively antivirulence therapy to prevent* P. aeruginosa* invasion holds strong grounds against development of bacterial resistance [15, 62]. The success of DhL against several virulence effects of* P. aeruginosa* in this study can lead to new treatment strategies and could also pave the way to the reduction of the amount of antibiotics required.

## Figures and Tables

**Table 1:** Minimum inhibitory concentration against *P*. *aeruginosa* strains was carried out previously [33]^*∗*^. It shows complete killing (MIC)^*∗*^, 50% of killing (MIC_50_)^*∗*^, and 90% of killing (MIC_90_)^*∗*^.

*P*. *aeruginosa* strain	Antimicrobial agent	MIC	MIC_90_	MIC_50_
		(mg/mL)	(mg/mL)	(mg/mL)
PAO1	DhL	0.48	0.24	0.12
	Gentamycin	0.075	0.064^*∗*^	0.032^*∗*^

PA14	DhL	0.96	0.48	0.24
	Gentamycin	0.1	>0.064^*∗*^	>0.032^*∗*^

PA103	DhL	0.48	0.24	0.12
	Gentamycin	0.075	0.064^*∗*^	0.032^*∗*^

CDN118	DhL	0.98	0.56	0.28
	Gentamycin	0.1	>0.064^*∗*^	>0.032^*∗*^

**Table 2:** Bacteriocidal (+) and bacteriostatic (−) effect of DhL on several experimental strains of *Pseudomonas aeruginosa*.

*Pseudomonas aeruginosa* strains	Bacteriocidal (+)	Bacteriostatic (−)
PAO1	+	
PA103	+	
PA14		−
Clinical strain		−

Bacteriocidal (MBC/MIC = 1 or 2).

Bacteriostatic (MBC/MIC = 4 or 16).

Results are means of number of colonies ± standard deviation.

**Table 3:** FICa: MIC of the combination/MICa alone. FICb: MIC of the combination/MICb alone. FICI: FICa + FICb. FICI interpretation: synergistic effect when FICI ≤ 0.5, additive effect when 1 > FICI < 0.5, and antagonistic effect when FICI > 1.

Microorganism	MIC (*μ*g/mL) Chloramphenicol	FICa	FICb	FICI	Effect
*Pseudomonas aeruginosa* PAO1	>400	0.25	≤0.03	≤0.28	Synergistic

## References

[1] Mesaros N., Nordmann P., Plésiat P. (2007). *Pseudomonas aeruginosa*: resistance and therapeutic options at the turn of the new millennium. *Clinical Microbiology and Infection*.

[2] Gomes M. Z. R., Machado C. R., De Souza da Conceição M. (2011). Outbreaks, persistence, and high mortality rates of multiresistant *Pseudomonas aeruginosa* infections in a hospital with AIDS-predominant admissions. *Brazilian Journal of Infectious Diseases*.

[3] Engler K., Mühlemann K., Garzoni C., Pfahler H., Geiser T., Von Garnier C. (2012). Colonisation with *Pseudomonas aeruginosa* and antibiotic resistance patterns in COPD patients. *Swiss Medical Weekly*.

[4] Hogardt M., Heesemann J. (2013). Microevolution of *Pseudomonas aeruginosa* to a chronic pathogen of the cystic fibrosis lung. *Current Topics in Microbiology and Immunology*.

[5] Wisplinghoff H., Seifert H., Tallent S. M., Bischoff T., Wenzel R. P., Edmond M. B. (2003). Nosocomial bloodstream infections in pediatric patients in United States hospitals: epidemiology, clinical features and susceptibilities. *Pediatric Infectious Disease Journal*.

[6] Kessler E., Safrin M., Abrams W. R., Rosenbloom J., Ohman D. E. (1997). Inhibitors and specificity of Pseudomonas aeruginosa LasA. *The Journal of Biological Chemistry*.

[7] McIver K. S., Kessler E., Ohman D. E. (2004). Identification of residues in the *Pseudomonas aeruginosa* elastase propeptide required for chaperone and secretion activities. *Microbiology*.

[8] Lee V. T., Smith R. S., Tümmler B., Lory S. (2005). Activities of *Pseudomonas aeruginosa* effectors secreted by the type III secretion system in vitro and during infection. *Infection and Immunity*.

[9] Hauser A. R. (2009). The type III secretion system of *Pseudomonas aeruginosa*: infection by injection. *Nature Reviews Microbiology*.

[10] Livermore D. M. (2001). Of Pseudomonas, porins, pumps and carbapenems. *Journal of Antimicrobial Chemotherapy*.

[11] Nordmann P., Guibert M. (1998). Extended-spectrum beta-lactamases in *Pseudomonas aeruginosa*. *Journal of Antimicrobial Chemotherapy*.

[12] Micek S. T., Lloyd A. E., Ritchie D. J., Reichley R. M., Fraser V. J., Kollef M. H. (2005). *Pseudomonas aeruginosa* bloodstream infection: importance of appropriate initial antimicrobial treatment. *Antimicrobial Agents and Chemotherapy*.

[13] Hauser A. R., Sriram P. (2005). Severe *Pseudomonas aeruginosa* infections. Tackling the conundrum of drug resistance. *Postgraduate Medicine*.

[14] Hentzer M., Wu H., Andersen J. B. (2003). Attenuation of *Pseudomonas aeruginosa* virulence by quorum sensing inhibitors. *The EMBO Journal*.

[15] Adonizio A., Kong K.-F., Mathee K. (2008). Inhibition of quorum sensing-controlled virulence factor production in *Pseudomonas aeruginosa* by south Florida plant extracts. *Antimicrobial Agents and Chemotherapy*.

[16] Choi E., Park H., Lee J., Kim G. (2013). Anticancer, antiobesity, and anti-inflammatory activity of *Artemisia* species in vitro. *Journal of Traditional Chinese Medicine*.

[17] Kim J.-H., Jung S.-H., Yang Y.-I. (2013). Artemisia leaf extract induces apoptosis in human endometriotic cells through regulation of the p38 and NF*κ*B pathways. *Journal of Ethnopharmacology*.

[18] Kalemba D., Kusewicz D., Świa̧der K. (2002). Antimicrobial properties of the essential oil of *Artemisia asiatica* Nakai. *Phytotherapy Research*.

[19] Ramezani M., Fazli-Bazzaz B. S., Saghafi-Khadem F., Dabaghian A. (2004). Antimicrobial activity of four *Artemisia* species of Iran. *Fitoterapia*.

[20] Ahameethunisa A. R., Hopper W. (2010). Antibacterial activity of *Artemisia nilagirica* leaf extracts against clinical and phytopathogenic bacteria. *BMC Complementary and Alternative Medicine*.

[21] Giordano O. S., Guerreiro E., Pestchanker M. J., Guzman J., Pastor D., Guardia T. (1990). The gastric cytoprotective effect of several sesquiterpene lactones. *Journal of Natural Products*.

[22] Bohlmann F., Zdero C. (1972). Polyacetylenic compounds on the constituents of the tribe Arctotideae. *Chemische Berichte*.

[23] Guardia T., Guzman J. A., Pestchanker M. J., Guerreiro E., Giordano O. S. (1994). Mucus synthesis and sulfhydryl groups in cytoprotection mediated by dehydroleucodine, a sesquiterpene lactone. *Journal of Natural Products*.

[24] Barrera P. A., Jimenez-Ortiz V., Tonn C., Giordano O., Galanti N., Sosa M. A. (2008). Natural sesquiterpene lactones are active against *Leishmania mexicana*. *Journal of Parasitology*.

[25] Vega A. E., Wendel G. H., Maria A. O. M., Pelzer L. (2009). Antimicrobial activity of *Artemisia douglasiana* and dehydroleucodine against *Helicobacter pylori*. *Journal of Ethnopharmacology*.

[26] Brengio S. D., Belmonte S. A., Guerreiro E., Giordano O. S., Pietrobon E. O., Sosa M. A. (2000). The sesquiterpene lactone dehydroleucodine (DhL) affects the growth of cultured epimastigotes of *Trypanosoma cruzi*. *Journal of Parasitology*.

[27] Setzer W. N., Vogler B., Schmidt J. M., Leahy J. G., Rives R. (2004). Antimicrobial activity of *Artemisia douglasiana* leaf essential oil. *Fitoterapia*.

[28] Priestap H. A., Abboud K. A., Velandia A. E., Lopez L. A., Barbieri M. A. (2011). Dehydro-leucodin: a guaiane-type sesquiterpene lactone. *Acta Crystallographica Section E: Structure Reports Online*.

[29] Rabe T., Mullholland D., van Staden J. (2002). Isolation and identification of antibacterial compounds from *Vernonia colorata* leaves. *Journal of Ethnopharmacology*.

[30] Berche P., Gaillard J.-L., Richard S. (1988). Invasiveness and intracellular growth of *Listeria monocytogenes*. *Infection*.

[31] Rosato A., Vitali C., De Laurentis N., Armenise D., Milillo M. A. (2007). Antibacterial effect of some essential oils administered alone or in combination with Norfloxacin. *Phytomedicine*.

[32] Wolfe E. J., Mathur V., Tomlanovich S. (1997). Pharmacokinetics of mycophenolate mofetil and intravenous ganciclovir alone and in combination in renal transplant recipients. *Pharmacotherapy*.

[33] Mahajan-Miklos S., Rahme L. G., Ausubel F. M. (2000). Elucidating the molecular mechanisms of bacterial virulence using non-mammalian hosts. *Molecular Microbiology*.

[34] Kessler E., Safrin M., Olson J. C., Ohman D. E. (1993). Secreted LasA of *Pseudomonas aeruginosa* is a staphylolytic protease. *The Journal of Biological Chemistry*.

[35] Bjorn M. J., Sokol P. A., Iglewski B. H. (1979). Influence of iron on yields of extracellular products in *Pseudomonas aeruginosa* cultures. *Journal of Bacteriology*.

[36] Livak K. J., Schmittgen T. D. (2001). Analysis of relative gene expression data using real-time quantitative PCR and the 2(-Delta Delta C(T)) method. *Methods*.

[37] O'Toole G. A., Kolter R. (1998). Initiation of biofilm formation in *Pseudomonas fluorescens* WCS365 proceeds via multiple, convergent signalling pathways: a genetic analysis. *Molecular Microbiology*.

[38] Jia J., Wang Y., Zhou L., Jin S. (2006). Expression of *Pseudomonas aeruginosa* toxin ExoS effectively induces apoptosis in host cells. *Infection and Immunity*.

[39] Hemaiswarya S., Kruthiventi A. K., Doble M. (2008). Synergism between natural products and antibiotics against infectious diseases. *Phytomedicine*.

[40] Hemaiswarya S., Doble M. (2010). Synergistic interaction of phenylpropanoids with antibiotics against bacteria. *Journal of Medical Microbiology*.

[41] Kyaw B. M., Arora S., Lim C. S. (2012). Bactericidal antibiotic-phytochemical combinations against methicillin resistant *Staphylococcus aureus*. *Brazilian Journal of Microbiology*.

[42] Jyot J., Balloy V., Jouvion G. (2011). Type II secretion system of pseudomonas aeruginosa: in vivo evidence of a significant role in death due to lung infection. *Journal of Infectious Diseases*.

[43] Toder D. S., Gambello M. J., Iglewski B. H. (1991). *Pseudomonas aeruginosa* LasA: a second elastase under the transcriptional control of lasR. *Molecular Microbiology*.

[44] Hoge R., Pelzer A., Rosenau F., Wilhelm S. (2010). Weapons of a pathogen: proteases and their role in virulence of *Pseudomonas aeruginosa*. *Current Reseach, Technology and Education Topics in Applied Microbiology and Microbial Technology*.

[45] Voynow J. A., Fischer B. M., Zheng S. (2008). Proteases and cystic fibrosis. *The International Journal of Biochemistry & Cell Biology*.

[46] Heck L. W., Morihara K., McRae W. B., Miller E. J. (1986). Specific cleavage of human type III and IV collagens by *Pseudomonas aeruginosa* elastase. *Infection and Immunity*.

[47] Costerton J. W., Lewandowski Z., Caldwell D. E., Korber D. R., Lappin-Scott H. M. (1995). Microbial biofilms. *Annual Review of Microbiology*.

[48] Davies D. (2003). Understanding biofilm resistance to antibacterial agents. *Nature Reviews Drug Discovery*.

[49] Schulert G. S., Feltman H., Rabin S. D. P. (2003). Secretion of the toxin ExoU is a marker for highly virulent *Pseudomonas aeruginosa* isolates obtained from patients with hospital-acquired pneumonia. *Journal of Infectious Diseases*.

[50] Holder I. A., Neely A. N., Frank D. W. (2001). Type III secretion/intoxication system important in virulence of *Pseudomonas aeruginosa* infections in burns. *Burns*.

[51] Moss J., Ehrmantraut M. E., Banwart B. D., Frank D. W., Barbieri J. T. (2001). Sera from adult patients with cystic fibrosis contain antibodies to *Pseudomonas aeruginosa* type III apparatus. *Infection and Immunity*.

[52] Kaufman M. R., Jia J., Zeng L., Ha U., Chow M., Jin S. (2000). *Pseudomonas aeruginosa* mediated apoptosis requires the ADP-ribosylating activity of ExoS. *Microbiology*.

[53] Stover C. K., Pham X. Q., Erwin A. L. (2000). Complete genome sequence of *Pseudomonas aeruginosa* PAO1, an opportunistic pathogen. *Nature*.

[54] Kukavica-Ibrulj I., Bragonzi A., Paroni M. (2008). In vivo growth of *Pseudomonas aeruginosa* strains PAO1 and PA14 and the hypervirulent strain LESB58 in a rat model of chronic lung infection. *Journal of Bacteriology*.

[55] Fleiszig S. M. J., Zaidi T. S., Preston M. J., Grout M., Evans D. J., Pier G. B. (1996). Relationship between cytotoxicity and corneal epithelial cell invasion by clinical isolates of *Pseudomonas aeruginosa*. *Infection and Immunity*.

[56] Schulert G. S., Feltman H., Rabin S. D. P. (2003). Secretion of the toxin ExoU is a marker for highly virulent *Pseudomonas aeruginosa* isolates obtained from patients with hospital-acquired pneumonia. *The Journal of Infectious Diseases*.

[57] Mikkelsen H., McMullan R., Filloux A. (2011). The *Pseudomonas aeruginosa* reference strain PA14 displays increased virulence due to a mutation in ladS. *PLoS ONE*.

[58] Ricardo Obando S. S., Jani M., Aguiar-Pulido V. Whole-genome assembly of multi-resistant *Pseudomonas aeruginosa* isolate.

[59] Siedle B., García-Piñeres A. J., Murillo R. (2004). Quantitative structure-activity relationship of sesquiterpene lactones as inhibitors of the transcription factor NF-kappaB. *Journal of Medicinal Chemistry*.

[60] Brutinel E. D., Vakulskas C. A., Brady K. M., Yahr T. L. (2008). Characterization of ExsA and of ExsA-dependent promoters required for expression of the *Pseudomonas aeruginosa* type III secretion system. *Molecular Microbiology*.

[61] Mathee A., von Schirnding Y. E. R., Levin J., Ismail A., Huntley R., Cantrell A. (2002). A survey of blood lead levels among young Johannesburg school children. *Environmental Research*.

[62] Clatworthy A. E., Pierson E., Hung D. T. (2007). Targeting virulence: a new paradigm for antimicrobial therapy. *Nature Chemical Biology*.

